# Gamified Versus Nongamified Metaverse Learning for Breast Health Knowledge in Women: Randomized Controlled Trial

**DOI:** 10.2196/75318

**Published:** 2026-02-13

**Authors:** Rui Li, Sabzali Musa Kahn, Bingyu Duan, Seng Yue Wong

**Affiliations:** 1 Dean Office, Academy of Malay Studies, Universiti Malaya, Kuala Lumpur, Malaysia, 60 379677245

**Keywords:** gamification, metaverse, health education, digital intervention, breast health knowledge

## Abstract

**Background:**

The metaverse provides an immersive, interactive medium for health education, but most studies evaluate immersion and gamification together. Randomized evidence disentangling their separate effects on immediate learning and short-term retention in breast health education is lacking.

**Objective:**

This study aimed to isolate the effects of gamification, over and above an identical immersive metaverse environment, on immediate gains and 4-week retention of women’s breast health knowledge.

**Methods:**

This 2-arm, parallel, individually randomized controlled trial was conducted in Hangzhou, China. Eligible participants were women aged ≥18 years who were interested in breast health and able to use a personal computer with internet access. A total of 80 women were recruited via the Xiaohongshu social media platform; 8 withdrew before randomization or did not complete the baseline assessment, and the remaining 72 women were randomized to a gamified metaverse (GM) group or a nongamified metaverse (NGM) group using a computer-generated 1:1 sequence. Both groups used the Mammoverse platform with identical educational content and exposure time. Breast health knowledge was assessed at baseline (T1), immediately postintervention (T2), and 4-week follow-up (T3) using the same questionnaire. The primary outcome was a change in knowledge score. Linear mixed-effects models were used, with age, education, family history of breast cancer, prior training, and baseline knowledge as covariates. Participants and investigators were not blinded.

**Results:**

All randomized participants completed follow-up and were included in the analysis (GM group: n=36; NGM group: n=36), with no loss to follow-up. Knowledge scores improved in both groups, but gains from T1 to T2 were larger in the GM group than in the NGM group (Hedges *g*=0.65, 95% CI 0.18‐1.12; *P*=.007). From T2 to T3, there was no between-group difference in change scores (*P*=.91). However, at 4 weeks, the GM group retained higher absolute knowledge than the NGM group (estimated marginal means 15.7 vs 13.0). No intervention-related adverse events were reported.

**Conclusions:**

This study marks the first application of gamification in breast self-examination education for ordinary Chinese women within a 3D desktop metaverse. By comparing gamified and nongamified versions under identical metaverse platform conditions, it expands the application boundaries of the GM group in breast health education. Gamification significantly enhanced immediate acquisition of breast health knowledge but did not provide additional advantages for short-term retention. However, the gamified group maintained higher absolute knowledge levels at the 4-week follow-up. Overall, in the 3D desktop metaverse, immersive experiences provide foundational effects, while gamification delivers immediate gains. To further optimize long-term retention, memory consolidation strategies should be integrated into the gamified framework.

## Introduction

Digital health interventions have emerged as a vital approach for health education and behavior change [1]. In recent years, immersive technologies such as the metaverse have garnered significant attention for their ability to deliver heightened presence and interactivity, thereby effectively addressing the limitations of traditional educational models in practical application and contextual learning [2]. In medical education, metaverse technology has demonstrated reusable learning benefits and accessibility advantages in anatomy and clinical skills training [3-5]. It also offers new implementation pathways for public health education, focusing on privacy protection, remote accessibility, and resource equity [6,7].

Meanwhile, gamification, as an effective motivational strategy, enhances engagement and behavioral adherence through designs incorporating points, goals, feedback, leaderboards, and story-driven tasks. In health and medical education, gamification has repeatedly been shown to improve short- and medium-term memory retention as well as learning engagement [8,9]. However, evidence regarding its long-term effects remains inconsistent. Some studies indicate that purely competitive gamification designs may induce learning anxiety [10,11] or fail to effectively enhance long-term retention due to concerns about privacy, team collaboration, and technical barriers [12]. These phenomena collectively suggest that gamification effects may primarily amplify immediate learning drive, whereas long-term knowledge consolidation likely relies more on other cognitive mechanisms.

Although immersive technologies and gamified designs are often integrated in practice, research on the medical metaverse has primarily focused on specialized domains such as clinical training and surgical simulation [13-15], with limited systematic validation for public-facing breast health education targeting women. Enhancing breast health information literacy and knowledge is a critical prerequisite influencing women’s screening intentions and health decisions [16]. However, existing digital interventions in this field are predominantly limited to two-dimensional web pages, minigames, or conceptual explorations [17-19].

More importantly, most studies combine immersion and gamification into a single integrated intervention or compare them only with traditional teaching methods, making it difficult to isolate the independent contributions of each mechanism [20-22]. Therefore, when observing positive effects on knowledge enhancement, a critical question arises: to what extent should these gains be attributed to the immersive experience provided by the metaverse, and to what extent to the motivational mechanisms of gamification? Distinguishing the unique contributions of different design elements is crucial for optimizing intervention efficiency, precisely controlling development costs, and guiding future personalized recommendations.

Based on this research, although a published mixed methods study has preliminarily confirmed the tool’s feasibility and potential motivational mechanisms, the attribution of its positive effects remains unclear [23]. Therefore, this study was designed as a randomized controlled trial (RCT). By introducing an NGM control group, it aims to systematically isolate the unique contributions of immersion and gamification effects to answer the core question: does a gamified intervention in a metaverse environment significantly outperform a metaverse-only environment in enhancing and sustaining breast health knowledge?

This study proposes the following 2 hypotheses:

- H1: In immediate post-intervention measurements, the enhancement of breast health knowledge delivered by the GM group will be significantly greater than that of the NGM group.
- H2: In delayed measurements, the retention effect of breast health knowledge in the GM group will be significantly superior to that of the NGM group.

The unique contributions of this study are as follows: (1) conducting an element-level causal test of immersion versus gamification within the same metaverse platform; (2) distinguishing and quantifying immediate and retention effects using linear mixed models (LMMs) and estimated marginal means (EMMs); and (3) providing replicable digital intervention evidence for public health scenarios such as women’s breast health education, along with design strategies for knowledge retention.

## Methods

### Study Design

This was a 2-arm, parallel-group RCT with 1:1 allocation. Participants were randomly assigned to 2 groups: the experimental group received a gamified health intervention within the metaverse (GM group), whereas the control group used a nongamified version of the same metaverse platform (NGM group). To assess both immediate and sustained effects of the intervention, 3 measurement points were established: preintervention (T1), immediate postintervention (T2), and delayed postintervention (T3), conducted 4 weeks after the intervention.

**Figure 1.**
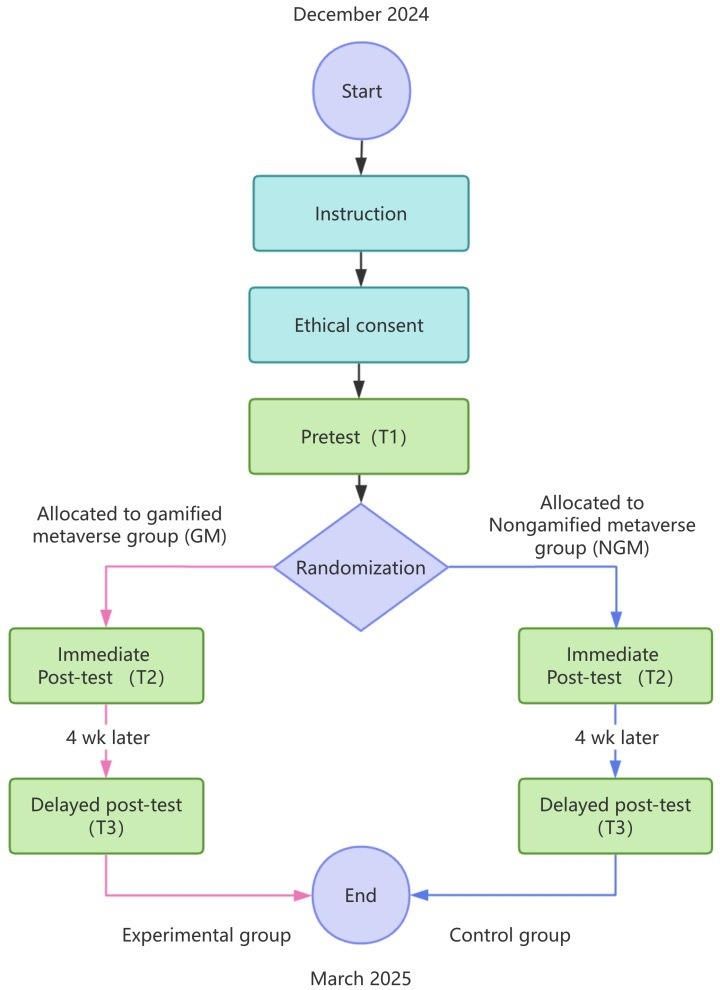
Study procedure and timeline.

This study adhered to the CONSORT (Consolidated Standards of Reporting Trials) 2025 statement (Checklist 1) and the CONSORT-EHEALTH (Consolidated Standards of Reporting Trials of Electronic and Mobile Health Applications and Online Telehealth, version 1.6.1) guidelines (Checklist 2) to enhance the reproducibility and transparency of research on web-based and digital health interventions [24,25]. In accordance with these guidelines, the study was retrospectively registered at ClinicalTrials.gov (NCT06930898). This study did not involve patient or public participation in the design, implementation, or reporting of research findings. Although a small number of participants self-reported having breast-related health issues, they were included as members of the general adult population rather than as patient representatives or research collaborators. Throughout the trial, all participants received the same intervention procedures and measurement schedules, and no significant modifications were made to the study procedures or outcome indicators based on interim results. The overall study flow is illustrated in Figure 1.

### Setting

Participant recruitment and data collection were conducted online in Hangzhou, Zhejiang Province, China. The intervention was delivered through a custom-developed 3D platform named “Mammoverse,” which integrates a metaverse-based immersive learning environment with gamified design principles.

The platform employs a semi-immersive 3D scene presentation mode, in which users navigate using a keyboard and mouse to complete learning activities, including exploration, tasks, quizzes, and feedback. This level of immersion strikes an optimal balance between information delivery and ease of interaction, enhancing participant focus while effectively reducing cognitive load, thereby supporting long-term knowledge retention [26]. Given the advantages of personal computer (PC) devices in stability and controllable interaction, the intervention was implemented remotely via a web-based platform, allowing participants to complete all learning and assessment procedures in their home or study environments.

### Participants

Participants were recruited using a combination of snowball and convenience sampling to ensure sample feasibility and diversity. Researchers posted recruitment notices on Xiaohongshu, where eligible and willing individuals could contact the research team via private message, complete an application form, and provide basic demographic information. To broaden coverage, the recruitment process allowed participants to invite friends or family members to join.

A total of 72 eligible participants completed baseline measurements and were randomized. Researchers completed the study using the online random number generation platform Research Randomizer [27]. After researchers set parameters as 2 groups, 36 participants per group, ID range 1 to 72. The platform automatically generated 2 nonoverlapping random ID sequences, participants were sequentially assigned to either the GM intervention group or the NGM control group based on the system output. Because the randomization sequence was automatically generated by the online system, researchers cannot predict subsequent allocation outcomes prior to assignment. Grouping operations were then performed based on the pregenerated sequence. As the 2 intervention modes were presented as distinct buttons on the platform interface, participants could identify their assigned intervention group upon accessing the platform; therefore, blinding could not be implemented for either participants or intervention implementers.

Given the increasingly younger age of individuals at risk for breast health issues and the rising probability of such risks with age, the inclusion criteria for this study were as follows: (1) women aged 18 years or older; (2) interest in breast health topics; (3) ownership and proficiency in operating a PC, mouse, keyboard, and stable internet connection; and (4) voluntary participation in the study and signing of an electronic informed consent form. Exclusion criteria applied to participants who were unable to meet any of the above requirements.

To ensure sufficient statistical power, this study conducted a pretest sample size estimation using G*Power version 3.1.9.6 software prior to implementation [28]. Based on a medium effect size (*f*=0.25), a significance level *α*=.05, and test power of 0.95, the analysis model was specified as a repeated measures design with a between-group factor (GM group vs NGM group) and a within-subject time factor (T1, T2, T3), allowing for the examination of the interaction between group and time. Calculations indicated a minimum sample size of 44 participants (22 per group) to meet the statistical requirements. Ultimately, 72 participants (36 per group) were recruited, significantly exceeding the minimum requirement. This larger sample size enhances the stability and generalizability of the findings. Furthermore, the sample size of this study aligns with that of previous RCTs in the same field, enhancing the comparability and external validity of the findings [29].

### Intervention

Both intervention conditions were based on the “Mammoverse” educational platform, an immersive 3D virtual clinic that enables users to interact with virtual characters and environments (Multimedia Appendix 1). This platform facilitates interactive learning through virtual characters within an immersive 3D virtual clinic setting. The overall design process of the platform has been systematically detailed in prior mixed methods research [23].

To examine the specific effects of knowledge enhancement, this study implemented 2 parallel intervention conditions. In the GM group, participants experienced the full gamified version described in the hybrid study [23]. This version employed a narrative-driven task flow to guide learning, where participants progressively mastered knowledge points through story-based missions. It also incorporated comprehensive incentive and feedback mechanisms, such as points, badges, and virtual rewards. In contrast, the NGM group used the same 3D educational content and scene structure but removed all gamification features and task guidance elements. In this version, participants could only freely explore the virtual clinic environment to acquire breast health knowledge, without narrative tasks or reward feedback mechanisms. Both intervention conditions were standardized in terms of platform configuration, interaction procedures, and delivery format, ensuring that the intervention protocols were fully replicable.

Both interventions ran on PCs using identical interaction methods, such as W/A/S/D keys for movement, the E key for interaction, and mouse operations. No harms or unintended effects were anticipated for either intervention. Participants were instructed to report any discomfort or adverse experiences during the study period. However, no such events were reported. Key functional differences between the 2 intervention conditions are summarized in Table 1.

**Table 1.** Functional comparison of the gamified and nongamified metaverse breast health education platforms.

Function	Gamified metaverse group	Nongamified metaverse group
Common core		
3D Immersive clinic	Yes	Yes
Core health knowledge content	Yes	Yes
Key differentiators		
Narrative context (story-driven experience)	Yes	No
Task-based process guidance	Yes	No
Points and rewards system	Yes	No
Badges	Yes	No
Learning modalities	Task-driven	Free exploration

### Instrument

The breast health knowledge questionnaire used in this study was adapted from the instrument developed by McCance et al [30]. It comprised 22 items organized into 4 sections: Section A collected demographic information (4 items); Section B assessed knowledge of breast cancer risk factors and screening (6 items); Section C assessed knowledge of breast self-examination (BSE) procedures and key techniques (10 items); and Section D assessed recognition of abnormal breast changes and symptoms (2 items). Excluding the demographic items, the remaining 18 items were single-choice or true or false knowledge questions. Each correct response was scored as 1, and each incorrect or “don’t know” response as 0, yielding a total knowledge score ranging from 0 to 18, with higher scores indicating greater breast health knowledge. Because all participants were native Mandarin Chinese speakers, the questionnaire was developed and administered in Chinese.

### Data Collection

The primary outcome measures of this study were the total scores for breast health knowledge at each time point and their changes relative to baseline. No secondary outcome measures were predefined. Therefore, data collection primarily centered on the 3 measurements of the breast health knowledge questionnaire. Participants first completed an online questionnaire primarily focused on breast health knowledge to assess their baseline knowledge level prior to the intervention. Immediately following the intervention, participants completed the same knowledge questionnaire as the pretest to evaluate the immediate impact on knowledge enhancement. Four weeks after the intervention concluded, all participants completed the same questionnaire again to assess the short-term retention of knowledge. All questionnaires were distributed online in PDF format, with participants submitting electronic versions through designated channels. The entire data collection process was remotely monitored by the research team to ensure completion. However, researchers did not provide any guidance or prompts during the questionnaire completion to guarantee data objectivity and consistency.

### Data Analysis

All data were analyzed using IBM SPSS Statistics (version 28.0; IBM Corp), with complete and missing-value-free sample data; thus, all randomly assigned participants were included in the primary analysis. First, descriptive statistics, including frequencies and percentages, were applied to participants’ demographic variables. Each item on the Breast Health Knowledge Questionnaire was scored as correct (1 point) or incorrect/unknown (0 points), with total scores at each time point representing knowledge levels.

To evaluate intervention effectiveness, LMMs were used to analyze score changes across groups and time points. The model included “knowledge score” as the dependent variable, with “group” (GM group vs NGM group) and “time” (T1, T2, T3) as fixed effects, and “participant ID” as a random effect to control for baseline differences between individuals. The primary focus was on examining the interaction effect between “group and time.” A significant interaction term (*P*<.05) would indicate statistically distinct trajectories of knowledge score change between the 2 groups. Post hoc comparisons of group differences were then conducted to further analyze score changes between T1 to T2 and T2 to T3, testing hypotheses H1 and H2. The above between-group difference comparisons represent exploratory post hoc analyses; this study did not prespecify or implement subgroup analyses or sensitivity analyses.

Before the model analysis, LMM assumptions were verified. The Q–Q plot indicated near-normal residual distribution, and the scatterplot of residuals versus predicted values showed no discernible patterns or heteroscedasticity, confirming good model fit (Multimedia Appendix 2). In addition to the prespecified intergroup comparisons described above, no subgroup analyses or sensitivity analyses were planned or performed in this study. All tests were 2-tailed, with a significance level set at *P*<.05.

### Ethical Considerations

This study was registered at ClinicalTrials.gov (NCT06930898). The study protocol and statistical analysis plan were reviewed and approved by the University of Malaya Research Ethics Committee (Ethics ID: UM.TNC2/UMREC_3967). The full protocol and statistical analysis plan are not publicly accessible but can be obtained from the corresponding author upon reasonable request. All study procedures adhered to the principles of the Declaration of Helsinki and the ethical guidelines for human subjects research.

Part of the analyses in this manuscript involved the use of deidentified data collected in a previous study conducted under the same ethics approval. In the original study, written informed consent was obtained from all participants, and the consent form explicitly stated that their data might be used for future related research. Therefore, the secondary analysis of the existing dataset fell within the scope of the original ethics approval, and no new informed consent or additional ethics review was required.

The study method adjustments implemented in this RCT, including the newly added randomization procedure and 4-week follow-up assessment, were reviewed and approved by the ethics committee as protocol amendments. For the RCT component of the study, all participants received full information about the study and electronically signed informed consent forms prior to participation.

All research data were deidentified prior to analysis and contained no personally identifiable information. Data were used solely for academic research purposes. Participants who completed all assessments received a compensation of 20 RMB (approximately US $2.8). All illustrative images, interface screenshots, and supplementary materials included in this manuscript contain no facial features or other identifiable personal information.

## Results

### Participant Flow and Recruitment

**Figure 2.**
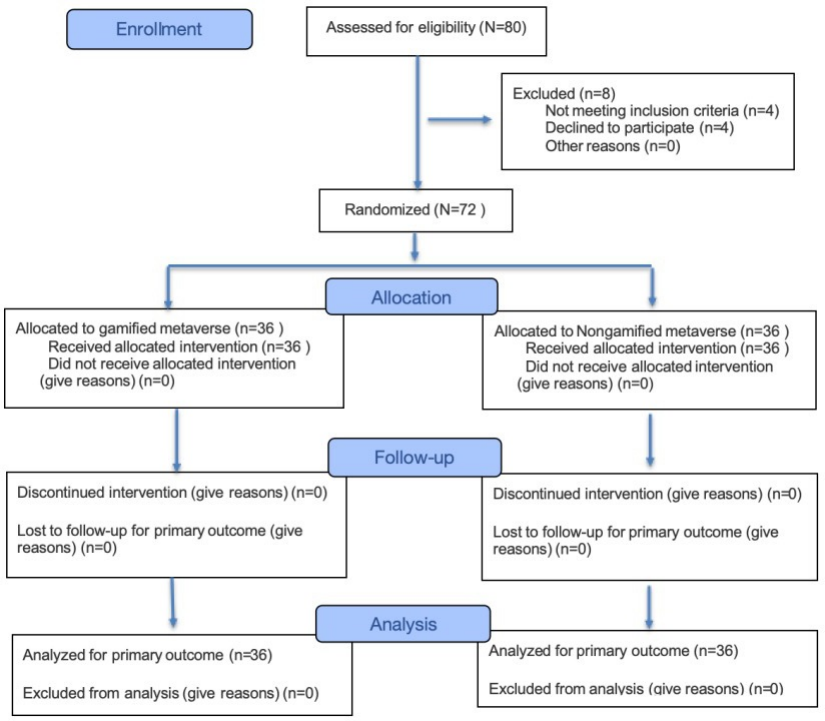
CONSORT (Consolidated Standards of Reporting Trials) 2025 flow diagram of participant recruitment, allocation, follow-up, and analysis.

The recruitment and follow-up process for this study were completed between December 2024 and March 2025. A total of 80 women underwent eligibility assessment, of whom 8 were excluded prior to random assignment: 4 did not meet inclusion criteria and 4 withdrew for personal reasons before completing baseline measurements. The remaining 72 eligible participants completed the T1 pretest and were randomly assigned to either the GM group (n=36) or the NGM group (n=36). All randomly assigned participants received the intervention according to their assignment and completed follow-up assessments. No participants were lost to follow-up or additionally excluded after randomization. Consequently, all 72 participants from both groups were included in the primary outcome analysis. Participant flow is shown in Figure 2.

### Intervention Delivery and Adherence

The intervention was implemented according to a predefined protocol. After all participants completed baseline testing, researchers sent remote access links based on random assignment. Participants then independently experienced the Mammoverse platform via PCs for approximately 20 minutes.

Prior to the formal intervention, researchers provided brief, standardized verbal instructions on basic operations, such as login procedures, interface navigation, and fundamental interactions, to ensure participants could successfully access the platform. Beyond these foundational operational guidelines, researchers did not engage in real-time instruction or intervene in participants’ learning content throughout the entire intervention period.

All randomly assigned participants received their respective interventions as per the study protocol. The intervention group completed gamified immersive learning tasks automatically presented by the platform, featuring incentive mechanisms such as points, badges, and leaderboards. The control group completed nongamified learning tasks identical in content to the intervention group but lacking gamification elements. The task flow and duration were identical for both groups. All participants completed all learning tasks required by the system prior to T2 (immediate test) and T3 (4 wk delayed test), with no dropouts or incomplete tasks, indicating good intervention adherence. During the study, no intervention-related adverse events or other harms were reported in either group. Neither group received additional educational materials or parallel interventions related to breast health, nor did they receive any concurrent care that could potentially affect learning outcomes, thereby ensuring the independence of the intervention effects.

### Baseline Characteristics

This study included 72 female participants who completed follow-up, with 36 in each of the GM group and the NGM group (Table 2). Both groups exhibited similar age distributions, with the majority aged 20 to 30 years (GM group: n=29, 80.56%; NGM group: n=28, 77.78%). Educational attainment was predominantly bachelor’s degree or higher: 61.11% (n=22) in GM group and 33.33% (n=12) in NGM group held bachelor’s degrees, while 52.78% (n=19) and 30.56% (n=11), respectively, held master’s degrees or higher. Most participants had no family history of breast cancer (n=31, 86.11% in both groups), and a low proportion had received breast health–related training (GM group: n=6, 16.67%; NGM group: n=4, 11.11%). Regarding baseline outcomes, T1 breast health knowledge scores were comparable between groups (GM group: mean 9, SD 2.6; NGM group: mean 8.3, SD 3.8), with a standardized difference of 0.20, indicating overall good balance after randomization. As preplanned, subsequent effect assessments will adjust for T1 as a covariate in the model.

**Table 2.** Baseline characteristics of women in the gamified and nongamified metaverse groups (N=72).

Variable	Gamified metaverse group (n=36)	Nongamified metaverse group (n=36)
Age (y), n (%)		
<20	2 (5.56)	5 (13.89)
20‐30	29 (80.56)	28 (77.78)
31‐40	3 (8.33)	2 (5.56)
>40	2 (5.56)	1 (2.78)
Education, n (%)		
Preuniversity	1 (2.78)	1 (2.78)
College	1 (2.78)	5 (13.89)
Undergraduate	22 (61.11)	19 (52.78)
Postgraduate and above	12 (33.33)	11 (30.56)
Family history of breast cancer, n (%)		
Yes	3 (8.33)	1 (2.78)
No	31 (86.11)	31 (86.11)
Uncertain	2 (5.56)	4 (11.11)
Education or training related to breast health, n (%)		
Yes	6 (16.67)	4 (11.11)
No	28 (77.78)	31 (86.11)
Uncertain	2 (5.56)	1 (2.78)
Baseline outcome, mean (SD)		
T1 breast-health knowledge^a^	9.0 (2.6)	8.3 (3.8)

a For T1, the standardized mean difference was 0.20; no significance test is performed. The primary analysis adjusted for T1 as a covariate.

### Immediate Knowledge Improvement: LMM Analysis

As shown in Table 3, the LMM revealed a significant main effect of group (*F*_1, 64.75_=29.110; *P*<.001), indicating that overall, GM group participants scored significantly higher on knowledge measures than NGM group participants. This establishes the overall superiority of the gamified intervention.

**Table 3.** Linear mixed-effects model of breast health knowledge scores by group and time.

Effect	*F* test (*df*)	*P* value	Interpretation
Group	29.11 (1, 64.75)	<.001	Overall higher knowledge scores in the gamified group
Time	212.72 (2, 113.69)	<.001	Knowledge scores increased significantly over time
Group×Time	5.53 (2, 113.69)	.005	The improvement pattern differed between groups (significant interaction)

The main effect of time was significant (*F*_2, 113.69_=212.70; *P*<.001), confirming that knowledge scores increased significantly over time during the intervention period (T1-T3), reflecting the effectiveness of the learning process.

The interaction between group and time was also significant (*F*_2, 113.69_=5.53, *P*=.005). This key finding indicates that the growth patterns of knowledge scores were not parallel across groups, with the gamified group exhibiting a significantly different improvement curve compared to the nongamified group. Therefore, we conducted between-group comparisons for T2-T1 and T3-T2 (see Table 4). Finally, after adjusting for age, education level, family history of breast cancer, prior training experience, and baseline (T1) scores, the primary findings remained consistent, with all *P*>.28 (Multimedia Appendix 3).

**Table 4.** Immediate knowledge improvement (T2-T1) and 4-week retention (T3-T2) in the gamified and nongamified groups.

Comparison	Group		Test^a^	Effect size^b^	*P* value	Interpretation
	GM^c^ (n=36)	NGM^d^ (n=36)				
T2-T1 (immediate improvement)	8.47 (2.5)^e^	6.25 (4.05)^e^	*t*_58.35_=2.8	Hedges *g*=0.65 (95% CI 0.18‐1.12)	.007	Greater improvement in gamified group
T3-T2 (retention)	36.22^f^	36.78^f^	*z*=−0.11	*r=*−0.02	.91	No difference in retention

a Two-sided *α*=.05.

b Effect size: Hedges *g* (for *t* test), *r* (for Mann-Whitney test).

c GM: gamified metaverse.

d NGM: nongamified metaverse.

e Mean (SD).

f Mean rank.

Table 5 presents the EMMs, showing significant improvements in both groups postintervention: GM group increased from 8.97 to 17.44, while NGM group rose from 8.33 to 14.58. At follow-up (T3), both groups experienced slight declines, but the GM group remained at 15.72, still higher than the NGM group at 12.97. Furthermore, the GM group maintained its advantage at both post-test and follow-up, particularly in timely improvement. The GM group (17.44) averaged approximately 3 points higher than the NGM group (14.58), indicating a greater enhancement. This suggests gamification delivers additional immediate gains within the same metaverse environment, supporting H1.

**Table 5.** Estimated marginal means (EMMs) of breast health knowledge scores by group and time^a^.

Group	T1 EMM (SE; 95% CI)	T2 EMM (SE; 95% CI)	T3 EMM (SE; 95% CI)
GM^b^	8.97 (0.41; 8.16‐9.78)	17.44 (0.41; 16.64‐18.25)	15.72 (0.41; 14.91‐16.53)
NGM^c^	8.33 (0.41; 7.53‐9.14)	14.58 (0.41; 13.78‐15.39)	12.97 (0.41; 12.16‐13.78)

a EMMs from the linear mixed model (restricted maximum likelihood estimation; Satterthwaite *df*); SEs are shown in parentheses; 2-sided *α*=.05.

b GM: gamified metaverse.

c NGM: nongamified metaverse.

### Knowledge Retention: Post Hoc Tests

Table 4 presents between-group comparisons for the enhancement and maintenance phases. For immediate improvement (T2–T1), Welch independent-samples *t* test was applied due to unequal variance (Levene *P*=.006). Results indicated that the GM group’s improvement (mean 8.47, SD 2.50) significantly exceeded the NGM group’s (mean 6.25, SD 4.05): *t*_58.35_=2.80, *P*=.007, with an effect size (Hedges *g*=0.65, 95% CI 0.18‐1.12), indicating a medium effect. This demonstrates that incorporating gamification elements within the same metaverse learning environment significantly enhances immediate knowledge acquisition, directly supporting H1.

The retention phase (T3-T2) employed the Mann-Whitney *U* test to compare knowledge retention or decline rates between groups: *z*=−0.11, *P*=.91, rank-biserial *r*=−0.02. Results indicated a negligible effect approaching zero, with no significant difference, suggesting comparable overall knowledge retention across groups during follow-up. Thus, H2 was not supported.

Thus, both groups gained learning benefits in the metaverse environment, but gamification delivered an additional, medium-sized immediate gain. Regarding follow-up retention, no significant intergroup differences emerged, indicating gamification’s limited impact on long-term retention.

## Discussion

### MainFindings

This RCT isolated and quantified the independent effects of immersive environments versus gamification mechanisms on breast health knowledge acquisition within the same Mammoverse platform. Under identical content and duration, the GM group significantly outperformed the NGM group only in “immediate enhancement,” supporting H1. However, it did not demonstrate superiority in “retention rate,” though scores at follow-up remained higher than the NGM group, failing to support H2. Despite comparable retention rates, the gamified group maintained higher knowledge levels at follow-up than the control (EMMs: 15.72 vs 12.97). These findings remained consistent after controlling for key covariates, including age, education level, family history of breast cancer, prior training experience, and baseline scores, further enhancing the robustness and reliability of the results.

From a mechanism perspective, both groups achieved significant improvements from T1 to T2. This finding strongly demonstrates that the metaverse scenario itself, as a highly immersive and interactive cognitive simulation environment, possesses fundamental pedagogical efficacy [31]. Building upon this foundation, gamification enhances short-term motivation and practice intensity through elements such as goal and task setting, scenario-based narratives, question-answer adjustments, and real-time feedback, thereby generating incremental effects during the immediate phase.

Based on H1, immediate postintervention measurements, the GM group significantly enhanced breast health knowledge more than the NGM group. The gamified “incremental effect” likely enhances learners’ attention allocation and task persistence through goal-setting and immediate feedback, reducing ‘activation costs’ to generate greater knowledge gains in a short time. Long-term retention relies more on consolidation strategies like spaced repetition and retrieval practice [32]. This aligns with Self-Determination Theory’s fulfillment mechanisms of competence, autonomy, and relatedness, manifesting as greater immediate knowledge gains under identical content and duration. This “immediate gain” compounded with the “baseline gain” from the immersive environment resulted in more pronounced improvements at T2 [33-35].

The findings of this study regarding gamification’s “instant gratification” align closely with the widely recognized positive effects of gamification on short-term knowledge acquisition and motivation enhancement in existing literature. The findings support Haruna’s [9] observation that gamified platforms provide interactive and incentive mechanisms to effectively enhance students’ learning engagement and knowledge acquisition. This immediate boost confirms gamification’s role as a motivation-driven tool, significantly increasing user participation motivation and short-term focus through elements like points and rewards [36]. Particularly in health education, studies by Anderson, Romero-Alemán, Marcos, and others demonstrate that gamified interventions significantly improve participants’ cognitive understanding and short-term retention of health knowledge. This aligns with the greater immediate gains observed in breast health knowledge among the GM group in this study [11,37,38]. This finding reduces the “startup costs” of learning by satisfying competence and autonomy in Self-Determination Theory, thereby maximizing knowledge transfer in a short timeframe. This aligns with Wang et al’s [39] perspective that gamification enhances sustained engagement by influencing user perceptions. Although Tong and Hee [40] previously employed a two-dimensional online game for breast cancer education, this study demonstrated that gamified mechanisms (motivation-driven) layered upon an immersive environment (cognitive vehicle) yield significantly greater immediate knowledge gains than purely immersive learning when comparing GM group with NGM group. This effectively distinguishes the critical contributions of immersion versus gamification.

Based on H2, delayed measurement revealed that the breast health knowledge retention effect in the GM group did not significantly outperform that in the NGM group. The “baseline gains” observed in the immersive metaverse may stem from heightened presence and actionable contextual practice. On one hand, the 3D interactive environment and actionable contextual practice significantly enhanced learners’ sense of presence, facilitating the formation of embodied cognition [41]. On the other hand, immersive experiences and immediate visual feedback help generate richer contextual encoding cues in the brain, effectively promoting active knowledge processing [42]. In other words, the immersive metaverse provides a high-fidelity cognitive vehicle for content learning, ensuring learners achieve significant knowledge gains with equivalent content and time investment [43].

The findings on long-term knowledge retention in this study provide new empirical evidence for understanding the cognitive vehicle role of immersive technologies (metaverse) and the long-term limitations of gamification. The GM group’s knowledge retention did not significantly outperform the NGM group, supporting existing literature indicating limitations of pure gamification mechanisms in long-term knowledge retention [8,10]. This suggests that while gamification’s immediate incentives can enhance short-term focus, its effects on intrinsic motivation and long-term memory consolidation are inferior to consolidation strategies like spaced repetition or retrieval practice [44]. Sestino et al [12] also noted that the long-term efficacy of gamification remains to be fully validated. The primary contribution of this study lies in identifying the foundational role of the metaverse environment in knowledge retention, rather than gamification mechanisms. The similar long-term retention performance observed in both the GM and NGM groups strongly supports the notion that the metaverse itself serves as an effective cognitive carrier. This finding resonates with conclusions from Werner, Iwanaga, and others regarding virtual reality (VR)’s enhanced learning outcomes in medical training through contextual simulation and 3D interaction, emphasizing embodied learning’s role in consolidating complex knowledge [4,5]. The heightened sense of presence and interactive contextualized practice provided by immersive environments helps form more robust memory anchors in the brain, thereby effectively promoting active knowledge processing and long-term retrieval [45].

This study confirms that the immersive metaverse serves as an efficient vehicle for foundational gains, providing robust support for learning. Gamification, building upon this foundation, generates immediate gains primarily by amplifying present learning motivation to achieve a cumulative effect. It is particularly suitable for health education scenarios requiring rapid acquisition of core knowledge, such as campus outreach, introductory courses, mobile microlearning, and resource-constrained environments. Designs should prioritize combining immersive environments with gamification to enhance immediate effectiveness. When targeting retention, reinforce this framework with consolidation modules such as spaced repetition, retrieval practice, delayed feedback, periodic recall challenges, and personalized reminder notifications. This transforms instant gains into sustained advantages.

### Limitations

It is important to emphasize that this study did not directly measure potential mediating variables such as motivation, self-efficacy, or cognitive load. Consequently, it was not possible to directly test the mediating role of “motivation or immersion.” Therefore, future research could incorporate such variables to examine mediating effects, thereby providing more causal empirical evidence regarding the contribution pathways of gamification and metaverse environments to learning outcomes under identical conditions. Second, the follow-up period in this study was limited to 4 weeks, restricting the assessment of longer-term, sustained knowledge retention effects. The existing data cannot determine the Mammoverse platform’s resistance to knowledge forgetting over months or even half a year. Additionally, this study was registered retrospectively after the trial commenced. Although the study content and procedures fully align with the ethical approval documentation, this procedural factor has somewhat limited the transparency of the research report and should be noted when interpreting the results. Concurrently, minor protocol amendments were implemented during the study period with ethics committee approval. None of these amendments involved core elements such as recruitment criteria, intervention content, or sample size. Consequently, they are not anticipated to substantially impact the study’s internal validity. In summary, this study robustly confirms that GM group environments significantly enhance short-term knowledge acquisition and sustain high follow-up retention levels. However, to substantially improve long-term knowledge retention rates, future instructional designs must strategically integrate cognitive-based science memory consolidation modules onto existing motivational frameworks.

### Innovation and Contribution

In terms of innovation, past explorations in breast health have primarily focused on patient education, lacking research aimed at public awareness dissemination, and employing relatively limited approaches. Existing studies have either examined gamification applications in breast health, such as online breast cancer education games, serious games for self-advocacy and emotional coping, computerized brain training games, step-counting gamified interventions, and breast cancer diagnosis simulation games, to enhance patients’ breast cancer knowledge, emotional regulation, and lifestyle [40,46-50]. Alternatively, research focuses on metaverse applications in breast health, including metaverse-based genetic counseling panels for breast cancer, augmented reality-assisted breast surgery education and remote guidance, young women’s demand for peer support in the metaverse, digital twin breast cancer models, and privacy-preserving metaverse medical data platforms for breast cancer [17-19,51,52]. However, no studies have yet combined gamification with metaverse platforms for BSE education. This research represents the first empirical study on GM group applications in breast health education, embodying its core innovation at the applied level.

Compared to existing research, GM group studies in recent years have predominantly utilized head-mounted display VR [53-55], or mobile augmented reality as primary platforms [22,56,57], while explorations specifically using 3D desktop metaverses as platforms remain relatively scarce. Existing 3D desktop studies either employ observational designs or single-group pre-post comparisons without control groups [58-60] or contrast 3D desktop environments with traditional lectures or case-based learning [29,61]. Furthermore, explorations of these GM groups in health education have largely been absent in mainland China and predominantly focused on nursing [62,63], predominantly utilizing VR-based approaches. Unlike previous studies, this research establishes both gamified and nongamified versions within the same 3D desktop metaverse platform, conducting an RCT with ordinary women in mainland China. This expands the application scope of gamified metaverses and introduces novel dissemination methods for breast health education and BSE knowledge promotion. Furthermore, prior research has indicated that superficial gamification elements yield no additional benefits in highly immersive VR environments [64]. However, this study identified significant differences within a semi-immersive desktop environment, validating the effectiveness of GM group platforms for public education on breast health knowledge.

In the real world, against the backdrop of an increasingly younger onset of breast cancer, this study demonstrates that a high-quality immersive 3D desktop metaverse serves as an efficient and scalable platform for breast health education. When resources are limited, and the primary goal is knowledge dissemination, it can serve as a foundational platform for hospital health screening centers, community initiatives, and online programs. By incorporating gamification elements such as tasks, points, and rewards in moderation, engagement levels and short-term learning outcomes can be significantly enhanced. At the same time, the findings remind us that relying solely on one-time gamified experiences is insufficient to ensure long-term knowledge retention and consistent BSE behavior. In real-world practice, task design must be integrated with actual self-examination behaviors. Overall, this study demonstrates that the 3D desktop metaverse possesses a degree of universality and equity across different age groups, educational levels, and risk exposure populations, providing a viable reference for future digital health education initiatives.

### Conclusions

As a gamified learning platform based on a metaverse environment, Mammoverse demonstrates clear benefits in enhancing women’s breast health knowledge. Under conditions of equivalent content and learning duration, the GM group achieves greater immediate knowledge gains compared to the NGM group. While retention rates were similar between groups during follow-up, the gamified group maintained higher absolute knowledge levels at the four-week mark, indicating its comprehensive advantage in both short-term learning outcomes and sustained retention. To focus on long-term retention, integrating memory consolidation modules into the existing framework is recommended to convert immediate advantages into sustained retention. Mammoverse offers a viable pathway for health education. By introducing a structured perspective that positions “immersion as a foundational gain and gamification as an immediate gain” into public health education, this study provides empirical support for promoting efficient, scalable breast health interventions in resource-constrained and mobile environments.

## Supplementary material

10.2196/75318 Multimedia Appendix 1 Screenshots of the Mammoverse intervention application interface illustrating key user interactions.

10.2196/75318 Multimedia Appendix 2 Linear mixed model diagnostics for the primary outcome: standardized residual Q–Q plot (A) and residuals versus fitted values (B).

10.2196/75318 Multimedia Appendix 3 Sensitivity analyses for the primary outcome: type III tests of fixed effects from the adjusted model (dependent variable: T2_3score).

10.2196/75318 Checklist 1 CONSORT checklist.

10.2196/75318 Checklist 2 CONSORT-eHEALTH checklist (V 1.6.1).

## Abbreviations

BSE: breast self-examination
CONSORT: Consolidated Standards of Reporting Trials
CONSORT-eHEALTH: Consolidated Standards of Reporting Trials of Electronic and Mobile Health Applications and Online Telehealth
EMM: estimated marginal mean
GM: gamified metaverse
LMM: linear mixed model
NGM: nongamified metaverse
PC: personal computer
RCT: randomized controlled trial
