# Supplementary figures and images for: Gamified Versus Nongamified Metaverse Learning for Breast Health Knowledge in Women: Randomized Controlled Trial
Source: JMIR Serious Games. 2026 Feb 13;14:e75318. doi: 10.2196/75318 (PMC12904354; doi:10.2196/75318)

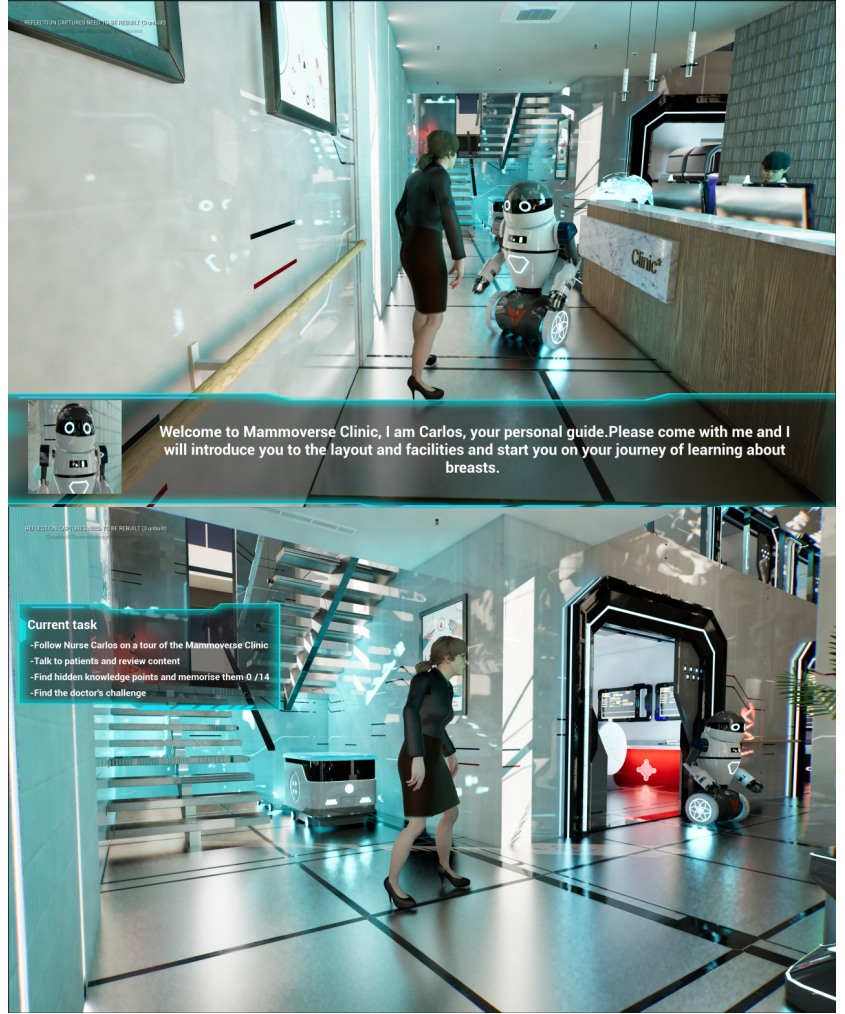

Supplement: Multimedia Appendix 1 [file games-v14-e75318-s001.png]

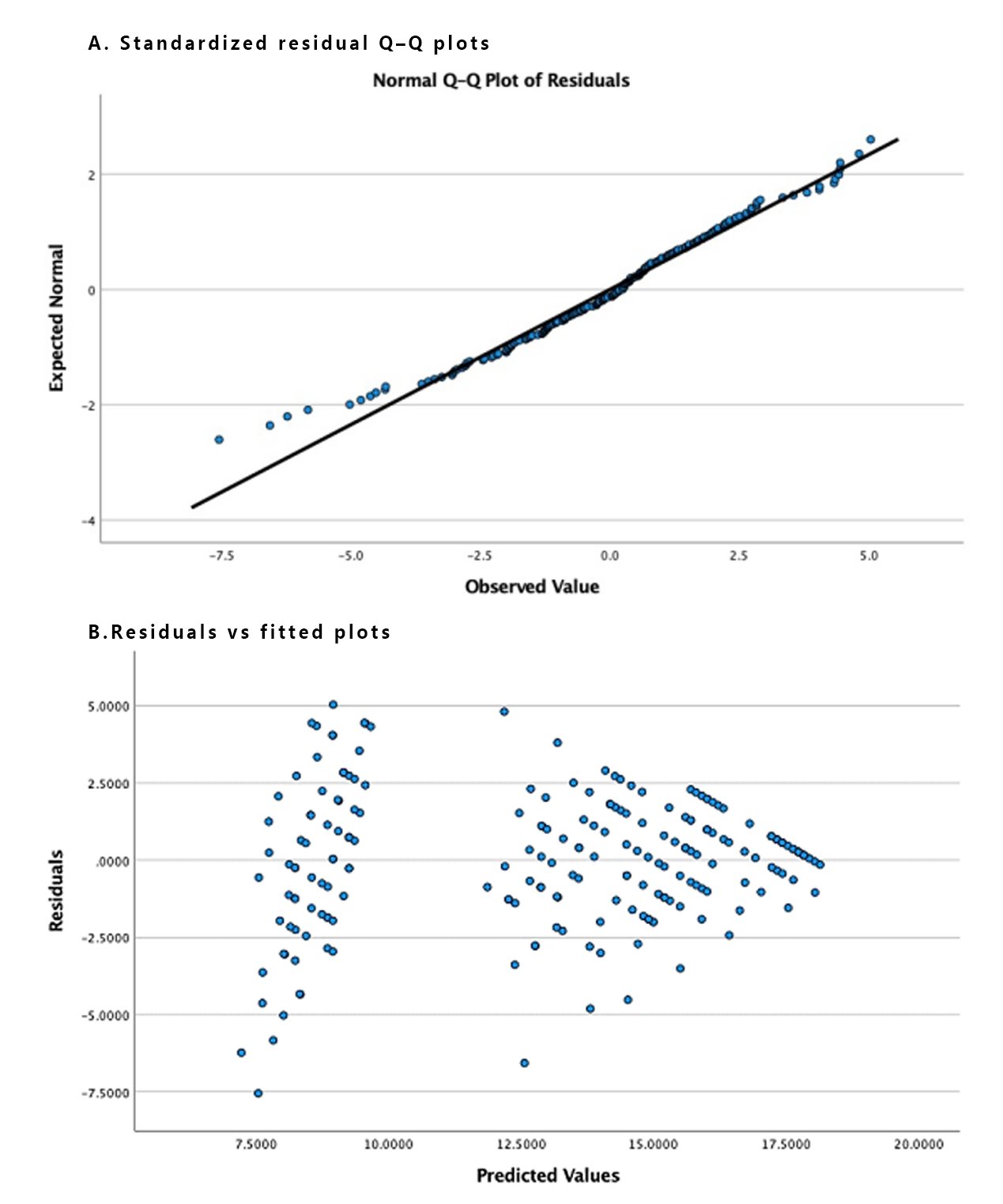

Supplement: Multimedia Appendix 2 [file games-v14-e75318-s002.png]

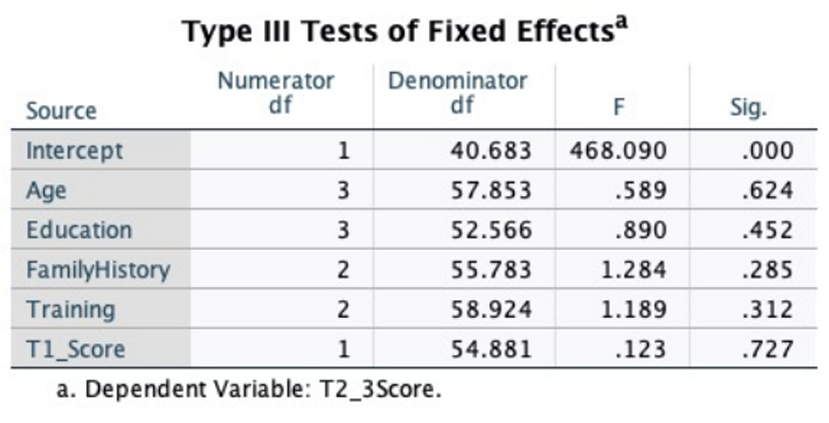

Supplement: Multimedia Appendix 3 [file games-v14-e75318-s003.png]
